# Extracellular Vesicles in Viral Liver Diseases

**DOI:** 10.3390/v16111785

**Published:** 2024-11-17

**Authors:** Elias Kouroumalis, Ioannis Tsomidis, Argyro Voumvouraki

**Affiliations:** 1 Laboratory of Gastroenterology and Hepatology, University of Crete Medical School, 71500 Heraklion, Greece; itsomidi@gmail.com; 2 1st Department of Internal Medicine, AHEPA University Hospital, 54621 Thessaloniki, Greece; iro_voum@yahoo.gr

**Keywords:** extracellular vesicles, exosomes, miRNAs, hepatitis B, hepatitis C, liver fibrosis, hepatocellular carcinoma

## Abstract

Extracellular vesicles (EVs) are bilayer vesicles released by cells in the microenvironment of the liver including parenchymal and non-parenchymal cells. They are the third important mechanism in the communications between cells, besides the secretion of cytokines and chemokines and the direct cell-to-cell contact. The aim of this review is to discuss the important role of EVs in viral liver disease, as there is increasing evidence that the transportation of viral proteins, all types of RNA, and viral particles including complete virions is implicated in the pathogenesis of both viral cirrhosis and viral-related hepatocellular carcinoma. The biogenesis of EVs is discussed and their role in the pathogenesis of viral liver diseases is presented. Their use as diagnostic and prognostic biomarkers is also analyzed. Most importantly, the significance of possible novel treatment strategies for liver fibrosis and hepatocellular carcinoma is presented, although available data are based on experimental evidence and clinical trials have not been reported.

## 1. Introduction

Many viruses can cause inflammation in the liver. However, the term viral hepatitis refers to five viruses that have the liver as the main target, although symptoms from other systems may also occur. These viruses are known under the first five letters of the alphabet as hepatitis A, B, C, D, and E (HAV, HBV, HBC, HBD, and HEV, respectively). With the exception of HAV, the incidence rates of HCV and HEV infection have not considerably changed worldwide between 1990 and 2019. An increased incidence in HAV infection has been observed in adults in the US, Europe and Asia. The overall incidence of HEV is lower than that of HAV in population studies, but the incidence of symptomatic acute icteric hepatitis cases of HEV is two times higher than the number of HAV cases. Moreover, HEV is associated with higher mortality. The incidence of acute hepatitis B has declined, due to safer medical practices and the wider application of HBV vaccinations. In 2020, HBV- and HCV-related diseases were responsible for approximately 1.1 million deaths. The death rate was roughly similar to tuberculosis and almost doubled compared to HIV or malaria. Africa and the South East Asian regions have a high incidence of HBV infections but 57% of the global mortality occurs in the Western Pacific region [1,2,3].

Chronicity is associated with HBV, HCV, HDV, and rarely with HEV. HAV does not progress to chronic disease. The absolute number of chronic viral liver cases (at any stage of disease severity) is over half a billion worldwide, but this may be an underestimation as accurate data are not available from countries like China and India [4].

During the last twenty years, the pathogenesis of viral liver disease has been focused on the communication of the complex network of cells in the liver microenvironment. Extracellular vesicles (EVs) have emerged as critical cell-derived particles implicated in acute and chronic liver injury irrespective of etiology [5]. EVs are important mediators of cell-to cell communication by delivering significant biological cargos [6]. EVs from damaged hepatocytes and liver sinusoidal endothelial cells (LSECs) activate the hepatic stellate cells (HSCs) and induce their proliferation [5]. EV release increases after liver injury [7,8,9].

Extracellular vesicles (EVs) are very small membrane-bound structures produced by all types of liver cells and secreted into the extracellular matrix [10]. They transfer nucleic acids, proteins, lipids, viral elements, and even complete virions among cells [11]. Viruses exploit and manipulate the EV biogenetic pathway, thereby increasing their ability to replicate, disseminate, and evade the attack by the immune system. EV is a general term that includes exosomes, microvesicles, and apoptotic bodies. They are collectively referred to as extracellular vesicles due to the difficulty in their separation and isolation. Exosomes are implicated in the pathogenesis of viral liver disease by modulating the host innate and adaptive reactions [12]. On the other hand, it should be noted that the exosomes derived from liver immune cells participate in antiviral immune defense and the elimination of hepatitis viruses [13,14]. As important transporters between cells, EVs may be used as prognostic and therapeutic agents, in addition to their role in pathogenesis. A better understanding of the mechanisms of EV involvement will lead to a better comprehension of viral pathogenesis and possibly to new diagnostic and therapeutic approaches [15].

Therefore, this review mainly summarizes the role of exosomes in viral hepatitis and its associated liver diseases, aiming to provide new strategies for the clinical treatment of liver diseases. It should be stressed that the majority of studies refer to exosomes and they are reported as such in this review. Some studies refer to EVs without distinctions. We included these as they include exosomes and microvesicles and we thought that the end result would be interesting.

## 2. An Overview of Extracellular Vesicles

### 2.1. Extracellular Vesicles (EVs)

Extracellular vesicles are bilayer vesicles released from the cells of most living organisms including plants [16]. However, their composition is extremely variable and this heterogeneity is responsible for their differential structure and functions. The protein composition is very similar to the plasma membrane (PM) of the producing cell [17]. The released EVs contain all kinds of non-coding RNAs, such as microRNAs (miRNAs), long non-coding RNAs (lncRNAs), and other ncRNA species such as PIWI interaction RNAs (piRNAs), circular RNAs (circRNAs), and transfer RNAs (tsRNAs) [18]. Under physiological conditions, they serve as the intercommunication between various cells; however, in pathological circumstances, they participate in the induction, aggravation, and persistence of several inflammatory diseases including viral liver diseases [19].

The accumulation of ILVs leads to the generation of multivesicular bodies (MVBs). The exact pathways of MVB formation have not been totally clarified [20,21]. After their generation, MVBs follow one of two possible ways. Either they are fused with the PM or they are fused with lysosomes for degradation [22]. In the first case, the small GTPases, Rab27a and Rab27b, assist MVBs to dock in the PM in those destined to secretion. MVBs then attach to soluble N-ethylmaleimide-sensitive protein receptors on the PM [23,24], and ultimately, they are released into the extracellular matrix (ECM) after fusion with the PM [25,26]. The decisive factor that determines the fusion with either the PM or lysosomes has not been fully investigated. It seems, however, that the final pathway is determined by the alternative action of inhibitors on either pathway as the inhibition of lysosomes leads to increased EV release [27,28]. Moreover, the biogenesis of EVs is also regulated by several additional factors such as G protein-coupled receptors that assist in the sorting of protein, lipids, and RNA cargos to obtain a precise biochemical content [29,30,31].

EVs are generally divided into the following two categories according to their biogenesis: exosomes and ectosomes. Exosomes are EVs produced from endosomes and released as previously described. Their production relies on the endosomal sorting complex required for transport (ESCRT) pathway. On the other hand, ectosomes are produced by the budding and blebbing of the PM. However, many ectosomes carry endosomal elements. Ectosomes include small-sized microvesicles (MVs), medium-sized microvesicles, and larger-sized apoptotic bodies [32,33].

Therefore, a uniformly accepted classification of EVs recognizes exosomes that are 50–150 nm in diameter, microvesicles that are 100–1000 nm in diameter, and apoptotic bodies that are 50–4000 nm in diameter [34,35]. Exosomes carry surface markers such as CD9, CD63, and CD81 of the tetraspanin family, that may distinguish them from microvesicles and apoptotic bodies [36]. Despite the existence of these markers, the differentiation of exosomes from MVs is not always possible due to the overlap in size and other physical characteristics. Although it is necessary to study these two types separately, as far as possible, the International Society for Extracellular Vesicles (ISEV) advocates the use of the generic term “extracellular vesicle” (EV) [17,37].

Normal cells produce exosomes and MVs. On the contrary, apoptotic bodies are only produced from cells undergoing apoptosis, as their name denotes. Apoptotic cells are also capable of releasing smaller apoptotic bodies that are sometimes called apoptotic microvesicles [38]. The clinical significance of apoptotic bodies was only recently recognized in full, but the relative research is still preliminary in contrast to exosomes and MVs [39].

### 2.2. Exosomes

Exosomes were first described in reticulocytes in the early 1980s. The first function attributed to exosomes after their discovery [40,41] was the removal of useless cellular proteins. Exosomes were named by Johnstone (1987) [42].

Exosomes are either small (60–80 nm) or large (90–120 nm) with a different consistency [43,44]. Small exosomes contain endosomal proteins and phagocytic vesicles originating in the endosomal compartment. Large exosomes contain PM proteins, cell-linked proteins, and late endosomal proteins, indicating that they are possibly derived from the PM, and therefore represent atypical exosomes [45]. As mentioned before, the exosomal shell is not only related to the membrane of the cells of origin, but they are also equipped with additional recognition and stability-related molecules [46]. The cargo carried by exosomes consists of RNAs, lipids, and proteins to define their function. Exosomes, like any other EV, carry all kinds of RNAs, that are important target cell regulators [47].

Exosomes are produced by several cells such as fat, macrophages, dendritic cells, T cells, B cells, and stem and tumor cells, and can be found in every body fluid including blood, urine, and cerebrospinal fluid [48,49]. In particular, exosomes produced by plasmacytoid dendritic cells (pDCs) and other professional antigen-presenting cells (APCs), are transported to adjacent or distant tissues through blood circulation [50,51]. They are implicated in various important physiological and pathological processes including the modulation of diseases, cell migration, angiogenesis, immune response, and tumor cell growth [52,53].

The development of exosomes begins with the formation and accumulation of intra-luminal vesicles (ILVs) within the lumen of the acidic, endocytic MVBs. The early stages in exosome synthesis follow the synthesis of the intra-luminal vesicles (ILVs), which form by the inward budding of the endosome membrane that engulfs the cytoplasmic elements [54,55]. In the case of exosomes, two mechanisms are operative. The process is either regulated by the ESCRT proteins or by an ESCRT-independent mechanism which is mediated by membrane lipids and membrane-spanning proteins like tetraspanins [56]. In detail, the first mechanism involves ubiquitinated domains in exosome-specific proteins acting as the capturing sites for the ESCRT. ESCRT-0, ESCRT-I, ESCRT-II, and ESCRT-III comprise the ESCRT machinery necessary for sorting the ubiquitinated proteins into ILVs [38,57]. The initiating sorting event is the binding of the double zinc finger domain of the endosomal protein Hrs with phosphatidylinositol 3-phosphate (PtdIns-3-P), allowing for the binding of ESCRT-0 to the endosomal membranes and the eventual recruitment of the rest of the ESCRT cascade [58]. The MVBs then fuse with the PM and subsequently released as exosomes [59,60]. Exosomes then interact with the target cells through direct membrane fusion, micropinocytosis, or through receptor-mediated endocytosis [61]. Exosomes retain endosome-related proteins, such as annexins, lipid raft-associated proteins, and ESCRT proteins, which are used for their transport and fusion with the target cell [62]. They also express membrane proteins including tetraspanins such as CD63 and CD81, fusion proteins like CD9, the lysosomal protein Lamp2b, and heat shock proteins like HSP70 [63]. Tetraspanins play a vital role in mediating exosome formation, as mentioned above, as well as fusion with the recipient cells [62,64]. The role of the two small GTPases Rab27a and Rab 27b, involved in the regulation of the size of exosomes and the docking of the MVBs at PM, has been mentioned before [55,61].

The exchange of nucleic acids through exosomes was demonstrated in mouse and human mast cell lines. miRNAs and mRNAs were found in 1300 genes in exosomes which were not part of the producer cells and were considered as exosomal shuttle RNAs (esRNAs) that are delivered to a target cell, and were translated into functional proteins in the recipient cell [50]. Exosomes are not immunogenic which is a significant characteristic when it comes to therapeutic use [65]. They are also involved, among others, in tissue remodeling, transport of materials between cells, and metabolic regulation [26,66]. Importantly, they have been investigated in viral infections including hepatitis viruses [36]. Exosomes are stable structures, and their constituents are protected from degradation [67].

There are several suggestions concerning the importance of the individual elements of the exosomes. Studies have indicated that exosomes exert their regulatory role through internal microRNAs [68,69], but others have attributed their beneficial properties either to protein cargos [70], or to the lncRNAs [71].

### 2.3. Microvesicles (MVs)

Microvesicles (MVs) are a heterogeneous group of EVs. MVs are produced by the outward budding and pinching of the PM [58,72,73]. They exist in several sizes spanning from 100 nm to 1 μm in diameter and they contain a variety of bioactive elements [10,74]. The surface and luminal cargoes of MVs are heterogeneous according to the different cell types of origin or even according to different functional states of the same cell. Cells infected with viruses may secrete MVs containing viral proteins, RNAs and, in some instances, infectious virions. Autophagy may be involved in that process, since MVs isolated from infected cells were positive for LC3-II, which is an autophagy marker [74]. Membranes of MVs expose phosphatidylserine (PS) and contain other proteins, such as the matrix metalloproteinase MT1-MMP, two glycoprotein receptors (GP1b and GPIIb/GPIIa), the adhesion protein P-selectin, and the integrin Mac-1. Their lumen resembles that of the exosome in terms of the presence of particular cytosolic proteins. Heat shock proteins and several enzymes are present at concentrations analogous to those in the cytosol. Rho and Ras GTPases are involved in the regulation of the assembly of MVs cargo. As in exosomes, RNAs are also abundant, mostly miRNAs but also mRNAs and non-coding RNAs [75]. There are detailed catalogs on the components found in exosomes and MVs that are constantly updated as new information is provided, using as much as possible the standardized EV isolation protocols. Such a recently updated catalog is the compendium Vesiclepedia [76].

The molecular biogenesis of MVs has not been clarified. Their formation requires a complex redistribution of membrane lipids due to the action of many phospholipid transporters such as flippase, floppase, and scramblase [77]. ESCRT proteins such as the apoptosis-associated gene 2-interacting protein X (ALIX), TSG101, VPS22, and VPS4 are also involved in the generation of MVs [78,79]. MVs are formed in lipid-rich domains within the PM and are enriched in proteins and lipids including flotillin-1, integrins, and phosphatidylserine on their external bilayer [80]. MVs are released directly from the PM of cells by outward blebbing without a previous formation of MVBs [81]. Unlike exosomes, different small GTPases such as ARF1, ARF6, and RhoA are involved in the budding of MVs, particularly from the PM of tumor cells [78,82,83,84]. Microvesicles may initiate an acute inflammatory response or may modulate the immune response by transporting pro-inflammatory miRNAs and cytokines such as IL-1β [85].

### 2.4. Biomolecules Experimentally Identified in Liver-Derived EVs

The liver is the most important site of EV clearance. EVs accumulate in the liver of rodents approximately 10 min after intravenous injection and are retained there for hours or even days. The polarity of hepatocytes influences the uptake, the release of EVs, and their number and composition. There are differences between those handled in the apical side of the hepatocyte and those handled at the basolateral side facing the blood [86]. EVs are mostly scavenged by the Kupffer cells and to a lesser extent by the liver sinusoidal cells (LSECs) and hepatocytes [87]

EVs are produced from parenchymal and nonparenchymal liver cells and are involved in most liver diseases [88,89]. EVs derived from fat-laden hepatocytes induce the activation of hepatic stellate cells (HSCs) through the shuttle miR-128-3p which mitigates PPAR-γ expression [90,91]. On the other hand, LSEC-derived EVs induce HSC migration by activating the sphingosine 1-phosphate (S1P) signaling pathway in HSCs [92]. In addition, HSCs themselves release EVs. HSC-derived EVs contain connective tissue growth factor (CTGF) [93] and are enriched in platelet-derived growth factor receptor-α (PDGFRα), and thus promote the progress of liver fibrosis [94]. EVs from HSCs early in the activation process induce a proinflammatory phenotype in KCs due to the activation of the TLR4 signaling pathway on Kupffer cells. This effect is abolished in conditioned medium derived from HSCs at a later stage of activation [95].

The content of liver EVs is different according to their site of origin. The EVs from hepatocytes contain many proteins such as the asialoglycoprotein receptor, cytochrome P450 isoforms, and proteins of the complement system and coagulation pathway, as well as apolipoproteins, albumin, haptoglobin, and drug-metabolizing enzymes including uridine diphosphate glucuronosyltransferases (UGTs), alcohol dehydrogenase-1 (ADH1), and glutathione S-transferase (GST). They also contain several miRNAs such as miRNA-122, -192, and -128-3p [32,96,97]. Cholangiocytes contain polycystin-1 growth factors such as the fibroblast growth factor FGF7, the long ncRNA H19, and ligands such as the hedgehog ligands [98,99]. On the other hand, HSCs contain PDGFRα, hedgehog ligand Twist-1, CTG, miR-214, and the miR17-92 cluster [94,100]. EVs from LSECs contain SK1 and fatty acid-binding protein 4 [18]. The situation with Kupffer cells (KCs) has not been clarified. Kupffer cell-derived EVs have not been characterized, but these cells may well release EVs with a composition similar to those of other types of macrophages [101]. Recently, KCs in NASH models released endogenous exosomal miR-690 and shuttled this miRNA to hepatocytes, bone marrow recruited macrophages (BM), and HSCs. miR-690 directly represses fibrogenesis in HSCs, inflammation in BM macrophages, and de novo lipogenesis in hepatocytes [102]. Moreover, M2 macrophage-derived exosomal miRNAs contribute to pulmonary fibrosis [103].

The data from other systems indicate that exosomal miRNAs may also affect macrophage polarization. miR-32 stimulates M2 polarization [104]. miR-1246 favors M2 polarization by inhibiting NF-κB to activate the signal transducer and the activator of transcription 3 (STAT3) signaling pathway [105]. Similarly, miR-34a-containing exosomes derived from adipocytes suppress the expression of Krüppel-like factor (KLF) 4 to inhibit M2 polarization [106]. In contrast, miR-124-3p-containing exosomes derived from mesenchymal stem cells (MSCs) promoted M2 macrophage polarization [107]. Whether the same applies for Kupffer cells remains to be investigated.

## 3. EVs in Viral Hepatitis

EVs are central players in the pathogenesis of viral hepatitis as they are implicated in viral transmission, and the initiation of disease progression [108]. Moreover, the EVs derived from a variety of cells may modify the host immune response and manipulate the liver microenvironment [109,110,111].

Due to the existence of common pathways, there are similarities between the exosome biogenesis and the virus replication process. It is reported that viruses can hijack the exosome biogenesis machinery to their advantage in the assembly and egress from infected cells. Most importantly, they evade the immune cells response. Exosomes can transmit both complete virions and separate viral components or naked genetic materials [112,113]. The hijack of the ESCRT and the Rab-GTPase complex is used by viruses to release virions and enclose them in a lipid envelope (112). It should be noted that blocking the release of EVs reduces viral replication without affecting the viability of host cells [114]. MVBs are utilized by both enveloped viruses such as HBV [115], and non-enveloped viruses such as HAV and HEV [116,117].

Common characteristics between viruses and EVs are the reasons for viral success in sharing the same pathways. Their size is very similar, as the diameter of HAV is 30 nm and the diameter of HCV is 50 nm [63]. Many viruses are equipped with a membrane envelope rich in glycosphingolipids and cholesterol similar to that of EVs. Virions contain the complete genome of either DNA or RNA while EVs incorporate all kinds of nucleic acids, as mentioned before [50,118,119]. Another similarity between viruses and EVs is the presence of glycan molecules on their surfaces. Viral glycans such as blood antigen A originate from the host cells. Viruses use glycans to bind to cells or evade the immune system [120,121,122,123]. Moreover, EVs and viruses enter the target cells by endocytosis using either intercellular adhesion molecules (ICAMs) or receptors, and release their content into the cytoplasm [124].The complex network of the liver cells, on the other hand, may also recognize particles carrying viral antigens, and launch the immune responses of target cells to the virus [125]. It should be stressed that these similarities cause problems in the separation of virus-containing exosomes or microvesicles from the viral particles themselves. Therefore, the details of separation techniques should be scrutinized in every attempt to draw conclusions from the studies involving distinctions.

Hepatitis viruses do not replicate within non-parenchymal cells in the liver. However, viruses induce the intracellular expression of interferon I and III types and release anti-viral exosomes that stimulate the immune system against virus-infected hepatocytes [126]. EVs can promote or suppress viral infections. The regulatory mechanisms deciding the ability of EVs to favor or mitigate viral infections include the consistency of the viral cargo, the delivery of viral entry receptors to new cells, and the presence or absence of neutralizing antibodies in EVs. Innate immune reactions against hepatitis viruses is mainly dependent on natural killer cells (NK) cells, dendritic cells (DCs), and innate-like T cells. Exosomes produced by these immune cells carry either antiviral or immune inhibitory factors. NK cells release exosomes with a killing ability and antiviral proteins, such as CD56 and perforin. DC is the most effective APC against hepatitis viruses. Antiviral effects of EVs are also associated with the transport of host inhibitory factors, such as the apolipoprotein B mRNA editing enzyme catalytic subunit 3G (APOBEC3G), the interferon-induced transmembrane protein 3 (IFTIM3), and miRNAs that affect immunity to viral infections. EVs can also attach to virions and induce antiviral immunity by transporting viral pathogen-associated molecular patterns (PAMPs) to APCs, thus promoting the activation of NK cells and the proliferation of CD4+ and CD8+ T cells. This mechanism is appropriately known as the bait effect [13,127]. Moreover, Kupffer cells and BM-derived macrophages release exosomes carrying antiviral factors that are absorbed by diseased hepatocytes [126]. Macrophage-derived exosomes use the HAV receptor T cell immunoglobulin and mucin receptor 1 (TIM-1), to enter into the hepatocytes [128]. On the contrary, HCV and HBV increase mitophagy, a specialized form of autophagy, by inducing the translocation of Parkin to mitochondria. Mitophagy in infected hepatocytes ameliorates apoptosis, and thus favors the persistence of viral infection [129,130].

The influence of exosomes in hepatitis infection has some individual characteristics according to the responsible virus.

### 3.1. HAV and EVs

HAV is a positive-sense RNA virus with sporadic or epidemic occurrence. It is not associated with chronic persistent infection, but nonetheless is responsible for approximately 0.5% of deaths worldwide due to the development of acute liver failure (ALF). The consumption of food or water contaminated with the feces of an infected person or inadequate personal hygiene are responsible for the transmission of the virus [131].

HAV is classified as non-enveloped virus, but it has been demonstrated that quasi-enveloped Hepatitis A virions (eHAV) surrounded by a membrane are produced during the common use of the exosome biogenesis pathways [132]. The VP2 and VP1pX domains of the non-enveloped virion interact with several elements of the host ESCRT machinery such as ALIX, ESCRT-III, and VPS4 to become internalized into MVBs. The rich-in-lysine residues pX domain is an extension of the VP1 protein, and is not present on the surface of the naked virion [133]. It is responsible for the incorporation of the virion into MVBs [132,133,134,135,136]. The pX protein also interacts with ALIX to promote virion release through exosome-like single lipid bilayer vesicles [133]. eHAVs are EV-transmitted virions as they have a density of ~1.05–1.10 g/cm^3^, similar to that of exosomes. Additionally, eHAV-enriched fractions express the exosome markers CD9, CD81, and CD63 [137]. Moreover, the delivery of exosomes with viral RNA of HAV into the cytoplasm is mediated by the phosphatidylserine receptor HAV cellular receptor 1 (HAVCR1) and the cholesterol transporter Niemann–Pick disease, type C1 (NPC1), indicating that viral infection does not require an envelope glycoprotein [138]. eHAV membranes protect the virus from proteolytic activity, and the action of host-neutralizing antibodies [132,139]. The protective phenomenon is referred to as a Trojan exosome, in an analogy with retroviral infection [140,141] and it facilitates the virus spreading in the liver. These quasi-enveloped virions assist the transmission of HAV in other organs in addition to the liver spread. On the other hand, HAV virions coated with exosomes limit viral replication slowing the spread of HAV between hepatocytes [132].

### 3.2. HEV and EVs

HEV is also a positive-sense RNA virus. It is more frequently associated with ALF in pregnancy and in patients with an underlying liver disease. The estimated frequency of ALF may be as high as 4% among infected individuals [142,143]. A use similar to HAV of the ESCRT machinery seems to be necessary for HEV release through MVP, but the origins of HEV membranes are not fully clarified, although an origin from MVBs is possible [117]. HEV release is associated with a common pathway with exosomes [144]. Exosomes assist HEV in the evasion of the immune response, increase the cholesterol and phosphatidylserine levels, and promote HEV cellular uptake, its replication, and transmission [117,145]. Quasi-enveloped HEV (eHEV) are the dominant HEV particles that circulate in the blood, whereas non-envelope virions are prevalent in the feces [134]. Quasi-enveloped particles carry the phosphoprotein ORF3, which facilitates their egress from the infected cell and is not present in the naked virion [134,146]. ORF3 plays an important role protecting viral antigens from the action of neutralizing host antibodies [134]. Most of the results on the role of exosomes in HEV infection are based on rodent data, but similar mechanisms seem to be operative in humans [117]. An additional similarity with HAV is that eHEV binds to the cells less efficiently and requires a longer inoculation time to infect liver cells compared with non-enveloped HEV. Degradation of the eHEV membrane in the lysosomal compartment may augment its infectiousness [147].

### 3.3. HBV and EVs

#### 3.3.1. Viral Particles and EVs

HBV is a reverse-transcribing enveloped virus that binds to the sodium taurocholate co-transporting polypeptide (NTCP) of liver cells for entry. It affects more than 250 million chronic carriers and accounts for nearly 900,000 deaths per year, figures that are possibly underestimated. It is transmitted through blood, from mother to child, transfusions, liver transplantation, or sexual practices that favor contact with blood. HBV-related liver disease is a potential life-threatening condition due to the chronicity and progression to cirrhosis and hepatocellular carcinoma (HCC) [148]. The transmission of viral elements by EVs released from HBV-infected cells is the main mechanism that favors HBV spread. Proteomics have revealed that in humans, the protein consistency of EVs originating from HBV-infected cells is different compared to those released from healthy cells with higher amounts of complement component C9 (C9), and Von Willebrand factor, among others [149].

Exosomes from infected cells contain several HBV constituents, including viral proteins such as HBx, HBsAg, HBeAg, and HbcAg, viral DNA such as rcDNA and cccDNA, and viral RNA such as XBx RNA, HBs/p RNA, and HBV-miR-3 [150,151,152,153,154,155]. Importantly, it was recently determined that EVs secreted by HBV-infected cells also contain intact virions. The presence of the large Hepatitis B virus surface antigen (LHB) was also identified on the surface of the exosomes [156].

The HB virus exploits the trafficking pathway of the Rab family, in common with the non-enveloped viruses. The LHBs start the translocation of HBV from the endoplasmic reticulum (ER) to MVBs after interaction with Rab5B [157,158,159,160]. Within MVBs, the HBV uses Rab7a and Rab27 to assist its egress outside the PM [161,162]. Many other cellular elements such as ubiquitin-interacting adaptor γ2-adaptin and ubiquitin ligase Nedd4 mediate the final stages of viral assembly and EV-associated egress [158]. γ2-adaptin and Nedd4 interact with the HBV core and L envelope proteins modulating the transport of the viral constituents through the final endosomal pathway [163,164]. Mutations of host ESCRT-III and Vps4 mitigate the maturation of HBV, thus forming a detergent-resistant vesicle [158]. The entire virions are protected from host neutralizing antibodies by their inclusion within exosomes, due to the absence of Hepatitis B surface antigens on the outside of exosomes [151].

HBV-replicating cells also secrete large amounts of subviral particles assembled by the viral surface proteins, but lacking the full genome. They are presented as 22 nm spheres and filaments. Filaments carry a much larger amount of LHBs compared to spheres. Spheres are released via the normal secretory pathway, while viral particles and filaments are dependent on the ESCRT machinery and are released through the MVB pathway [165].

#### 3.3.2. EVs and Immune Regulation in HBV

Exosomes can modify the host immune response against HBV as they participate in mechanisms favoring the immune escape. Exosome-associated tetraspin CD63 is necessary for the efficient assembly of HBV. HBV viral particles that were released from CD63-depleted hepatocytes were significantly less infective than those released from control cells with normal CD63 levels [166]. Sera from patients with chronic HBV (CHB) contained HBV elements that inhibited the proliferation and survival of NK cells. In particular, HBV repressed the expression of the retinoic acid inducible gene I (RIG-I), on NK cells reducing the activation of the nuclear factor κB (NF-κB) and p38 mitogen-activated protein kinase pathways. The lytic activity of NK cells was diminished including a reduced production of IFN-γ and TNFa. Therefore, the replication and transmission of HBV was significantly increased [152]. Transcription products of the HBx gene were transported to the target cells through exosomes making the liver microenvironment favorable for HBV transmission [154].

Additional immunosuppressive mechanisms have been identified during CHB. An overexpression of miR-21 and miR-29 in exosomes from HBV hepatocytes reduced the production of IL-12 by dendritic cells and macrophages, leading to inactivation of NK cells, and impairment of the immune response [14]. The inhibition of the immune response has been verified in HBV transgenic mice, and the progression of CHB was promoted by HBV-derived exosomes due to the reduced clearance of HBV replication [153].

As mentioned before, EVs are not immunogenic by themselves. However, they can be taken up by immunocompetent cells when modified by the presence of HBV, and initiate an immune response. In vitro experiments showed that EVs produced by HBV-infected hepatocytes enter macrophages and induce the expression of a programmed cell death 1 ligand 1 (PD-L1) in monocytes in association with the downregulation of CD69 [167]. PD-L1 is a negative molecule that leads T cells expressing PD-1 to immune exhaustion, while CD69 is a marker of activated immune cells [168,169]. Similar findings with EVs released from cells with active HBV replication were described in vivo. They inhibited the eradication of HBV-infected cells in mice transfected with HBV. Moreover, these EVs were incorporated in organs outside the liver such as bone marrow and intestine. Importantly, bone marrow cells incorporating these EVs were accumulated in the intestine where the dendritic cell populations were substantially increased [170]. Humoral immunity also participates in the immune evasion of HBV. Neutralizing antibodies were also impaired with a low response to HBV-DNA in the presence of HBcAg+CD81+ exosomes. A similarly impaired response was observed in CD81+ hepatitis C virus (HCV)-associated exosomes indicating that the presence of CD81+ exosomes induce resistance to antibody neutralization and immune evasion of HBV [151]. Exosomal protein profiles are altered in HBV-infected cells. These alterations are implicated in immune regulation. Subunit proteins of the proteasome complex are selectively packaged into exosomes derived from replicating HBV cells leading to reduced production of cytokines such as IL-6 by human monocytes and impairment of the immune reaction [155].

In contrast to the studies above, several studies have reported that HBV-associated exosomes can have a different effect promoting the immune responses during HBV infection. EVs released by macrophages contain IFN-α-associated miRNA and exploit the virus entry pathway into infected hepatocytes. The RIG-I viral RNA sensor is activated by EV-HBV, and this leads to the induction of genes that belong to the INF family. RIG proteins compete with viral polymerases, and they block viral DNA replication in hepatocytes [128,171].

A recent experimental study reported significant differences in the profiles of proteins and host miRNAs between EVs derived from immortalized normal hepatocytes and HBV-infected HCC cell lines, emphasizing again the role of microRNAs in the evolution of HBV [172].

MicroRNAs are a class of non-coding RNAs approximately 22 nucleotides in length that target mRNAs for translational suppression [173]. miR-199a-3p and miR-210 significantly repressed the expression of hepatitis B surface antigen (HBsAg), and reduced HBV replication [174]. Exosomes produced from HBV-infected hepatocytes led to the upregulated expression of NKG2D ligands in macrophages, followed by induction of NK cells to produce IFN-γ in the early stage of HBV infection [14]. HBV-miR-3 is an HBV-encoded miRNA with anti-HBV activity. It was identified in HBV-infected hepatocytes and in the serum exosomes of patients with CHB. In hepatocytes, HBV-miR-3 targets a site of the HBV transcript to reduce the expression of HBc protein, pgRNA, and HBV replication intermediate (HBV-RI) levels without affecting the HBV DNA polymerase [175]. This miRNA downregulates the suppressor of cytokine signaling SOCS5 and activates the JAK/STAT pathway enhancing the antiviral effect of IFN. Exosomal HBV-miR-3 also promotes the M1 polarization of macrophages and increase IL-6 secretion [176]. On the contrary, opposite effects have been reported. miR-146a and FEN-1 present in EVs derived from HBV-infected cells inhibit the inflammatory function of M1 macrophages and promote immune evasion [177].

However, EVs from patients with CHB with persistently normal alanine aminotransferase levels and rich in miR-25-3p enhanced the proliferation of an HCC cell line indicating that EVs from infected HBV cells may promote the progression of liver cancer [178]. Similar conclusions regarding HBV-related HCC were also reported in another study. HBcore antigens upregulated the expression of exosomal miR-135a-5p. The analysis of patient samples revealed that miR-135a-5p was increased in HCC tissues in comparison with adjacent non-cancerous tissues. The target gene of miR-135a-5p was the vesicle-associated membrane protein 2 (VAMP2). Moreover, the exosomal miR-135a-5p inhibited apoptosis and promoted HCC cell proliferation and chemotherapy resistance [179]. Additionally, it was demonstrated both in vivo and in vitro that IFN-*α*-treated liver non-parenchymal cells showed an increased expression of antiviral proteins such as A3G and MyD88 in macrophages and LSECs. They were internalized by hepatocytes and suppressed HBV infection [126]. Macrophage-derived exosomes exploit the hepatitis A virus receptor TIM-1 to enter hepatocytes, as mentioned before. At a later stage, clathrin-mediated endocytosis is used by exosomes, followed by exosome–endosome fusion to transfer IFN-α-induced anti-HBV activity [128,180].

Some of the miRNAs enriched exosomes can impair the innate immune response by targeting pro-inflammatory cytokines. Exosomal miRNAs, such as miR-21, -192, -215, -221, and-222, derived from human hepatocytes infected with HBV, were found to target sequences of the 3′ untranslated region (UTR) of human IL-21 mRNA and suppressed the expression of IL-21 in human T helper 2 cells, a cytokine that is involved in anti-viral immunity [181]. The implication of the miR-222 and the transferrin receptor (TFRC) in liver fibrosis was investigated in exosomes rich in miR-222 derived from HBV-infected hepatocytes. These exosomes induced the activation of LX-2 cells (analogous to human HSC) and collagen production. This was associated with an inhibition of TFRC, indicating that miR-222 targets TRFC-induced ferroptosis which is a process that attenuates liver fibrosis [182]. Exosomes from pegylated IFN-α-treated responding patients, and the supernatants of IFN-α-treated macrophages showed anti-HBV activities, suppressing HBsAg, HBeAg, HBV DNA, and the covalently closed circular DNA (cccDNA) levels in HBV-infected cells. PegIFN-α treatment significantly increased exosomal miRNAs 193a-5p, -25-5p, and -574-5p, which could suppress HBV replication. No change in the three IFN-α-related miRNAs was observed in the IFN non-responders [183]. Overexpression of miR-3188 repressed HBV replication, while inhibition of miR-3188 increased its replication HBV. It was further demonstrated that the inhibitory effect of miR-3188 was mediated by targeting the host protein Bcl-2 [184].

In summary, exosomes may repress the antiviral immune responses mediating impairment of immune cells, including NK cells, PD-1-positive T cells, and DCs. They also protect HBV by inhibiting the action of HBV-neutralizing antibodies. On the other hand, exosomes may assist the antiviral immune responses. They activate macrophages and transport antiviral materials to hepatocytes to promote the immune response [185].

### 3.4. HCV and EVs

HCV is a positive-strand RNA virus of the Flaviviridae family. Infection occurs through exposure to blood from unsafe health care, unscreened blood transfusions, injection drug use, and sexual practices that lead to exposure to blood. An estimated 50 million people have chronic HCV, with approximately one million new infections occurring per year. In 2022, 242,000 people died from hepatitis C, mostly from cirrhosis and hepatocellular carcinoma [186]. As in HBV, these figures may indeed represent an underestimation, as in many countries, the statistics are probably not accurate.

Initially, it was believed that HCV was assembled in the ER and the virus was released through the common secretory pathway. However, it is now evident that the virus utilizes the exosomal pathway for both assembly and release [187]. It was demonstrated for the first time that exosomes could transmit HCV between hepatocytes [109]. Moreover, exosomes isolated from a cell line unable to produce intact virions due to the lack of necessary proteins were still able to infect other cells. In vitro exosomal transfer of HCV replicon led to less efficient cell infection [188]. EVs play an important role in carrying and transmitting HCV, including entire viral particles. Full length HCV RNAs are enclosed within hepatocyte-derived exosomes and can be transferred to plasmacytoid dendritic cells (pDC), where they induce IFN-α production [189]. Both structural and non-structural viral proteins are also transmitted by exosomes. Viral structural glycoproteins such as E1 and E2 have been identified in EV [190]. Syntenin-1, an intracellular adaptor protein, is implicated in the HCV envelope glycoprotein E2 enrichment of EVs [191].

EV encapsulation is as advantageous for the virus as it is for HBV. Efficient production of E2-coated exosomes protects HCV from antibody neutralization [191]. HCV can evade the immune system and can use additional receptors for entrance into the cells that are different from the viral receptors. Moreover, HCV can spread to organs outside the liver through exosomes [192]. Furthermore, HCV RNA can be stabilized by the binding to a series of proteins such as argonaut RISC catalytic component 2 (Ago2), heat shock protein 90 (HSP90), and miR-122 within EVs. Exosomes isolated from the serum of HCV-infected patients contain elevated levels of the proteins Ago2 and HSP90 in addition to HCV RNA. These exosomes could mediate viral receptor-independent transmission of HCV to hepatocytes. Interestingly, replication competent viral RNA was present in exosomes of all HCV patients non-responding to anti-viral treatment and in some treatment-naïve individuals [192,193].

**Figure 1:**
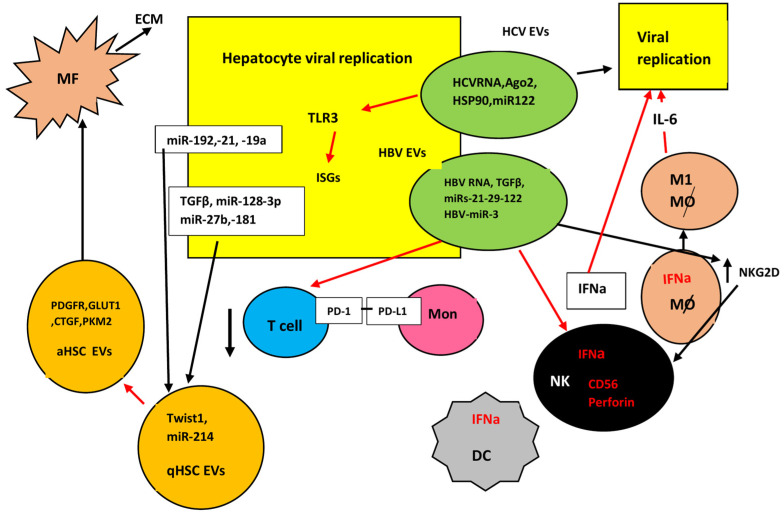
A simplified diagram indicating the roles of exosomes in viral hepatitis. HBV and HCV multiplied within the infected hepatocytes spread viral RNAs and miRNAs through exosomes to adjacent hepatocytes inducing further viral replication. At the same time, viral exosomes inhibit the defensive immune responses launched by immune cells against viruses. HCV-derived exosomes inhibit TLR3 of the hepatocytes reducing the expression of ISGs. In addition, exosomes from HBV lead to exhaustion of the T effector cells by upregulating PD-L1 expression in the surface of monocytes. On the other hand, HBV-miR-3 exosomes from HBV-infected hepatocytes activate NK cells to produce IFNa through upregulation of the NKGD protein in macrophages. Moreover, exosomes produced by either infected hepatocytes or viruses activate qHSCs into aHSCs. EVs produced by qHSCs are rich in miR-214, Twist 1, which attenuates the profibrotic function of activated HSCs, while exosomes from aHSCs contain CTGF and proteins that activate myofibroblasts to produce ECM or activate qHSCs. For more details, see text. MØ: macrophages; ISG: immune responsive genes; MF: myofibroblasts; DC: dendritic cells. Black arrows indicate activation. Red arrows indicate inhibition.

HCV-containing EVs also transport miRNAs such as miRNA-19a, miRNA-192 and miRNA-122. Additionally, cytokines and other factors assisting efficient viral replication have also been detected. HCV-EVs carry miRNA-192 that increases TGF-β production, promoting thus liver fibrosis [194]. EVs released by HCV-infected cells transmit anti-immunogenic signals. They induce RUNXOR lncRNA and RUNX-1 that upregulate many immunosuppressive molecules, such as arginase-1 (ARG1), inducible nitric oxide synthase 2 (iNOS), and STAT3. These EVs transport ROS molecules that can induce apoptosis in T cells [195,196,197]. Other miRNAs are also implicated in HCV infectivity. Umbilical MSCs-derived exosomes (uMSC-Exo) contained functional miRNAs, such as let-7f, miR-145, miR-199a, and miR-221 which contributed to the suppression of HCV RNA replication when released. These four miRNAs possessed binding sites in HCV RNA. EVs loaded with miR-199a or miR-221 repressed EV-associated HCV transmission to naive cells [198]. Similarly, to HBV, other EVs can stimulate the immune response. For example, HCV particles can be internalized by LSECs activating INF I and III production. Released EVs from these cells rich in INF proteins may block virus replication in the recipient cells [199]. A simplified diagram of exosomal functions is presented in Figure 1.

The critical role of EVs in the transmission of HCV is further supported by the studies in which the abolition of autophagy in HCV-infected hepatocytes reduced the incorporated HCV within EVs, resulting in the accumulation of virus particles within cells. An association between HCV and the exosomal marker CD63 was demonstrated in autophagy knockdown cells which may inhibit HCV assembly or release [200]. The impairment of autophagy in HCV-infected hepatocytes activates the interferon signaling pathway and induces apoptosis. Therefore, HCV-induced autophagy diminishes the innate immune response and promotes HCV replication [201]. There are additional mechanisms for the immune evasion of HCV assisted by exosomes. HCV forms a double-stranded RNA (dsRNA) intermediate during replication which is a major pathogen-associated molecular pattern triggering innate immune responses. Hepatocytes express several pattern recognition receptors (PRRs) for the detection of dsRNA, including the endosomal toll-like receptor 3 (TLR3) [202]. Exosomes containing HCV RNA reduce toll-like receptor 3 (TLR3) activation, inhibiting the production of IFN and IFN-stimulated genes (ISGs) [203]. The accumulation of Treg cells and increased inhibitory signaling pathways such as T-cell Ig and mucin domain protein-3 (Tim-3) and galectin-9 (Gal-9) repress the antiviral effector T cells that are essential for viral clearance. Similarly, monocytes incubated with EVs from HCV-infected hepatocytes increased their galectin-9 expression [204], which in turn has been shown to promote the expansion of regulatory T cells and apoptosis of HCV-specific T cells [205,206]. CD81 is a co-receptor of HCV entry into the cells and is enriched in exosomes. CD81+ exosomes carry HCV particles and lead to HCV immune evasion and persistence of HCV infection [207].

Interestingly, the role of plasma EVs from patients with HCV after treatment with direct-acting antiviral drugs (DAAs) was investigated. A significant reduction in miR204-5p, miR181a-5p, miR143-3p, and miR122-p in the EVs of HCV patients compared to healthy donors was established. Importantly however, EVs analyzed 6 months after DAAs, showed a persistence of low miR204-p and miR143-3p compared to healthy donors even after sustained virological response (SVR), indicating a possible link between EV-mediated signals and fibrosis progression even after SVR [208,209].

## 4. EVs as Markers of Viral Liver Disease

There is always a necessity to find suitable biomarkers capable of predicting the risk of viral hepatitis progression. The expression patterns of serum exosomal miRNAs have been used as potential biomarkers for the diagnosis and evolution of the grade and stage of viral liver diseases. Exosomal miRNAs are protected from degradation by exosomes in the extracellular environment [210]. Their stability, and wide distribution in body fluids, indicate that miRNAs may become novel diagnostic biomarkers for viral diseases [211].

Exosomes have been utilized for the prediction of HBV infection. HBeAg-positive patients had significantly increased plasma levels of miR-122-5p, -125b-5p, -192-5p, -193b-3p, and-194-5p compared to HBeAg-negative patients, and levels of these miRNAs were correlated to HBV DNA and HBsAg levels. More importantly, the plasma levels of miR-301a-3p and miR-145-5p were higher in responders compared to non-responders [212]. Moreover, serum miR-29, miR-143, miR-223, miR-21, and miR-374 levels were gradually reduced as fibrosis progressed from S0–S2 to S3–S4. A panel of three miRNAs and platelets could discriminate between S0–S2 and S3–S4, with high accuracy and an area under the curve of 0.936 [213]. The experimental evidence suggested that macrophage-derived exosomal miR-103-3p could promote HSC activation by targeting Krüppel-like factor 4 (KLF4). It was therefore implicated in the communication between macrophages and HSCs during the progression of liver fibrosis. As a biomarker of liver fibrosis in patients with HBV-related liver disease, it was accurately correlated with the degree of fibrosis [214].

An important observation for clinical practice was recently reported. In patients with CHB and negative for serum HBV-DNA, exosomal HBV-DNA was detectable and could be used to monitor the treatment effects. Exosomal HBV-DNA could be used in patients with a high suspicion of HBV infection who are negative for serum HBV-DNA [215], provided that a proper methodology for isolation and detection of viral exosomes is used [216]. Exosomal miRNAs are more sensitive to reflect liver inflammation activity than ALT [217], and exosomal miRNAs can be used as an auxiliary diagnosis of viral liver disease [218], particularly those with persistently normal ALT (PNALT). A study of ten patients with HBV-related histological inflammation and PNALT showed that 18 exosomal miRNAs were upregulated and 6 exosomal miRNAs were downregulated in PNALT patients with inflammation grade ≥ A2 compared with those with inflammation grade < A2 [219]. Serum exosomal miR-21 can be used as an accurate biomarker of liver injury. miR-21 is significantly higher in patients with CHB compared to healthy controls [220]. However, there are some problems with the use of exosomes as markers of CHB. None of the exosomal miRNAs identified so far is specific for the diagnosis of CHB. miR-21 may be a marker of non-HCC tumors such as colorectal cancer [221] and breast cancer [222] or of non-oncologic diseases such as type 1 diabetes [223]. Therefore, it can be used in HBV, but only if the co-existence of these other diseases has been excluded.

Proteomics of exosomes may be also useful as markers of HBV. The protein composition of exosomes derived from an HCC cell line infected with HBx was compared with uninfected cells. HBx overexpression caused significant changes in the protein composition of exosomes. Similar changes in protein contents were also identified in plasma exosomes purified from HBV-infected patients compared with plasma negative for HBV [224]. Recently it was reported that exosomes rich in albumin, vascular endothelial growth factor (VEGF), and CD63 were increased in CHB, but decreased in HBV-related acute-on-chronic liver failure (ACLF). Moreover, the same exosomes were significantly increased in the surviving patients compared with non-survivors. The sensitivity and specificity of these exosomes were higher than the other markers of liver regeneration. Exosomes rich in CD63 and albumin may be used as an early-warning marker in patients with ACLF [225]. In addition, the serum exosomal long-chain non-coding RNA nuclear-rich transcript 1 (lncRNA NEAT1) seems to predict the 90-day mortality in patients with HBV-related ACLF, better than MELD [226].

Circulating EVs are also used as markers in chronic HCV. Serum exosomal miR-122, -134, -424-3p, 629-5p, and -199a have been proposed as biomarkers for HCV [192,227,228] and exosomal miR-214 is a potential biomarker for liver fibrosis [229].

EVs derived from immune cells could differentiate patients with HCV from those with nonalcoholic steatohepatitis (NASH) [230]. The expression patterns of nine miRNAs identified patients with HCV and NASH with >95% accuracy and were correlated with the stage of liver fibrosis and the grade of liver inflammation in HBV. Moreover, the miRNA expression pattern of the early fibrotic stage was significantly different from that observed in high inflammation grades [231]. Levels of HCV-derived miR-19a exosomes correlated with liver fibrosis through a mechanism that was mentioned before [197]. miR-19a and miR-155 levels were correlated with advanced liver fibrosis in HCV patients. Moreover, the CD81 protein content of exosomes was positively correlated with the grade of inflammation and the severity of liver fibrosis [218,232]. Early-stage fibrotic HBV and HCV patients diagnosed by transient elastography were compared with normal controls. Circulating EVs with miRNA-192, -200b, -92a, and -150 were downregulated in both HBV and HCV patients, while miRNA-200b and miRNA-122 were increased in both conditions [233]. In chronic HCV, miR-21 expression was positively correlated with the viral load, fibrosis, and transaminase levels. On the other hand, miR-122 expression was negatively correlated with fibrosis, transaminase levels, and patient age. miR-21 enhanced the TGF-β signaling pathway, supporting the correlation between miR-21 and fibrosis [234]. In addition to exosomes, microvesicles have a potential to be biomarkers in viral liver disease. Thus, patients with chronic HCV infection secrete increased levels of microvesicles derived from CD4+ and CD8+ T cells compared with healthy individuals [230]. Similarly to HBV, proteomics of exosomes may be also useful as markers of HCV. Serum exosomal CD81 is increased in patients with chronic HCV, and its level correlates with the level of transaminases and severe liver fibrosis [232,235].

A particularly promising field is the application of EVs as potential markers of hepatitis-related HCC. Exosomal miRNAs have been tested for the differentiation among HCC and cirrhosis, CHB patients, or healthy controls. Several studies have proposed miR-21 as a biomarker for the early detection of HCC whether the initial factor was HBV or another etiology. Exosomal miR-21 was significantly elevated in patients with HBV-related HCC compared to patients with CHB or to healthy controls [220]. Plasma miR-21 levels were significantly reduced after radical resection in patients with HCC. Moreover, plasma miR-21 level was significantly increased in HCC compared to patients with chronic hepatitis and healthy controls. The sensitivity was 61% with an 83% specificity in differentiation of HCC from chronic hepatitis, while the sensitivity was increased to 87% with 92% specificity when differentiating HCC from healthy controls. In all groups, mir-21 values were superior to AFP, but the combination of AFP and mir-21 was superior as a discriminant marker than plasma miR-21 and AFP alone [236]. However, other studies of HBV-related HCC patients were unable to find a mir-21 difference among HCC patients and either CHB or HBV cirrhosis [237,238]. Different miRNAs were found to be related to HCC. Thus, miR-18a, -221, -222, and -224 were higher and miR-101, -106b, -122, and -195 were decreased in exosomes from HCC patients [237].

Other miRNAs have been tested in the diagnosis of viral-related HCC. A miRNA classifier (*Cmi*) containing miR-29a, -29c, -133a, -143, -145, -192, and-505) could accurately detect HCC. *Cmi* had a higher sensitivity than AFP in HCC detection, while its specificity was similar to AFP [239]. Exosomal miR-212 is increased in HBV-related HCC compared to HCC unrelated to HBV. Moreover, a linear correlation was observed between levels of mir-212 and the stage of HBV disease [240].

The circulating exosomal miR-125b levels were decreased in HCC patients compared with patients with CHB and cirrhosis [241]. Additionally, miR-125b levels were associated with tumor number, encapsulation, and TNM stage. HCC patients with lower miR-125b levels had a reduced time to recurrence and overall survival. These results indicated that exosomal miR-125b could be used as a prognostic marker for HCC [242]. Experimentally, miR-125b repressed HCC progression by inhibiting the epithelial–mesenchymal transition, migration, and invasion of hepatoma cells [243]. Serum exosomes hsa-circ-0028861 and hsa-circ-0070396 may be used as new biomarkers of HBV-related HCC [244,245]. Importantly, miR-125b-5p and -223-3p can also be used as biomarkers for HBV-related HCC at an early stage of CHB [246]. In HCV-related HCC patients followed for 10 years after radical resection, the level of circulating exosomal differentiation antagonizing non-protein coding RNA was positively correlated with HCC recurrence and was the best predictive factor of HCC recurrence and mortality [247]. A high expression of miR-483-5p in HBV-related HCC tumors was demonstrated in patients with short-term recurrence after resection of HCC. The activated leukocyte cell adhesion molecule (ALCAM), which is a suppressor of migration and invasion of HCC cells, was downregulated in those patients. Experimentally, it was shown that the direct target of mir-483-5p is ALCAM. These findings indicate that the exosomal miR-483-5p/ALCAM might be used as a marker of invasion and metastasis of HVB-related HCC. Further studies are required [248]. Interestingly, a prognostic exosome mRNA-related 4-gene signature model has been recently constructed and validated. A nomogram based on this model proved to have an ability for HCC [249].

The main EV markers are presented in Table 1.

## 5. EVs and Viral Fibrosis

### 5.1. A Brief Overview of Liver Fibrosis

Liver injury leads to hepatocyte damage with liver immune cell activation and liver infiltration by extra-hepatic immune cells. Quiescent HSCs (qHSCs) are activated and transformed into myofibroblasts, which are involved in tissue repair under normal conditions. In liver injuries of short duration, the pro-fibrotic and anti-fibrotic mechanisms of the liver are in balance, and fibrosis does not occur. However, when the injury is chronic, damaged hepatocytes release damage-associated molecular patterns (DAMPs). DAMPs, either directly or indirectly through liver macrophages, activate and maintain the fibrotic phenotype of HSCs which produce large amounts of ECM with type I and type III collagen and fibronectin as the main components. Activated HSCs release cytokines such as transforming growth factor β1 (TGF-β1), platelet-derived growth factor (PDGF), and connective tissue growth factor (CTGF). Autocrine secretions from activated HSCs (aHSC) continually activate qHSCs. DAMPs also induce the recruitment and activation of immune cells such as bone marrow-derived macrophages. These immune cells promote HSC activation and myofibroblast production by secreting pro-inflammatory and profibrotic factors [250,251,252].

Inflammation is the first response to hepatocellular death mediated by several inflammatory signals from various liver cell types. The extrahepatic mediators from the gut–liver axis are also involved. Single-cell technologies have revealed the heterogeneity of immune cells and the spatial organization within the liver, including Kupffer cells and recruited macrophages, neutrophils, effector and regulatory T cells, as well as innate lymphoid cells and unconventional T cell populations. In turn, aHSCs may modulate inflammation through chemokine and cytokine secretions [253].

The central point in the development of fibrosis is the HSC activation. Two main phases, initiation and perpetuation, are described. The initiation phase is an early change in gene expression. It is induced by DAMP signals from Kupffer cells, reactive oxygen species and lipid peroxide exposure, and extracellular matrix changes. The perpetuation phase involves the maintenance of the aHSCs ultimately leading to the development of fibrosis [254].

### 5.2. Role of EVs During HSC Activation

Exosomes are implicated in the pathogenesis of liver fibrosis, by regulating the activation of HSCs and the intercommunication between HSCs and immune cells. Hepatocytes-derived exosomes and HSC-derived exosomes may increase liver fibrosis, but most data are purely experimental and further research is necessary to prove that they are applicable to viral-induced cirrhosis [255,256]. Exosomes from various cells transport many regulator miRNAs, and new members are constantly described.

Exosomes derived from qHSCs, are kept to a basal level. Their exosomal cargo includes various transcription factors such as Twist 1, miRNAs such as miR-214, miR-199a-5 and proteins such as keratins and histones, that suppress fibrotic signals and maintain the balance between qHSCs and aHSCs phenotype [229]. However, opposite results were reported in a recent study in the bile duct-ligated model of fibrosis. HSC-derived exosomal miR-199a-5p, when transported to other HSCs and hepatocytes, promoted the epithelial–mesenchymal transition (EMT) and the senescence of hepatocytes. Furthermore, miR-199a-5p overexpression increased liver fibrosis in this rat model in parallel with SIRT 1 downregulation [257]. On the other hand, aHSCs are characterized by increased exosome secretion reflecting the phenotypical transformation into myofibroblasts. The cargo encapsulated in aHSCs exosomes consists of growth factors, miRNAs, and proteins such GLUT1 and PKM2 that are associated with the further activation of qHSCs and promotion of the fibrotic process [258].

Exosomes released by damaged HSCs are rich in fibrogenic contents, which can accelerate fibrosis and collagen deposition by liver myofibroblasts and fibroblasts [259,260]. CTGF, a multifunctional heparin-binding glycoprotein, is highly expressed in activated HSC-derived exosomes regulating the activation and migration of HSCs [93]. The induction of CTGF expression in HSCs by fibrotic stimuli was due to reduced expression of miR-214, a direct inhibitor of CTGF expression by binding to the 3′-untranslated region of CTGF RNA [261].

The experimental and clinical evidence indicate that PDGFRα is implicated in liver fibrosis. Patients with liver fibrosis had significantly higher PDGFRα levels in serum EVs compared to healthy controls. EVs derived from PDGF-BB-treated HSCs promoted HSC migration and liver fibrosis. Tyrosine720-to-phenylalanine mutation on the PDGFRα or inhibition of Src homology 2 domain tyrosine phosphatase 2 (SHP2) inhibited the enrichment of PDGFRα in EVs and directed the receptor to degradation. An inhibitor of SHP2 also attenuated liver fibrosis in the CCl4 mouse model [94].

HSCs at an early stage of activation trigger a proinflammatory phenotype in KCs via the release of EVs. This effect is absent at a later stage of activation and is dependent on the activation of the Toll-like receptor 4 (TLR4) in KCs. The phagocytic ability of Kupffer cells was not affected at any stage of HSC activation. This pro-inflammatory effect of HSCs is mediated through the secretion of EVs released from HSCs [95].

Exosomes from injured liver cells are rich in cytochrome P450. The reactive oxygen produced by cytochrome P450 2E1 (CYP2E1) produces the strong oxidant superoxide anion free radicals and hydrogen peroxide, among others, leading to hepatocellular apoptosis under pathophysiological conditions [5,262]. Hepatocyte lipotoxic fatty acid damage produces exosomes rich in miR17-92 clusters, which can be absorbed by HSCs, resulting in fibrotic activation [263]. Exosomes released from hepatocytes are implicated in the activation of HSCs. During HBV infection, exosomes derived from HBV-infected hepatocytes promoted HSC activation and fibrosis in a murine model. The exosomal miR-222 could enhance HSC activation by inhibiting transferrin receptor (TFRC)-induced HSC ferroptosis, as mentioned before [182].

Furthermore, exosomes produced by damaged LSECs are absorbed by adjacent fibroblasts, augmenting the production of a-smooth muscle actin and type I collagen to promote fibrosis [264]. In another study, EVs derived from LSECs were internalized by HSCs leading to sphingosine-1-phosphate-dependent HSC activation and promotion of liver fibrosis, as already mentioned above [92].

Moreover, HSC-derived exosomes stimulate the release of pro-inflammatory cytokines such as IL-6 and TNFa from macrophages, thereby increasing the macrophage inflammatory response and liver fibrosis [265,266]. Exosomes secreted by aHSC can further promote M1 polarization of macrophages, and the exosomal dihydrofolate reductase is involved in this effect [267]. Another important effect of exosomes released from aHSCs is the induction of glycolysis in hepatic non-parenchymal cells such as KCs and LSECs. The glycolysis-related proteins Glucose transporter type 1(GLUT1) and Pyruvate kinase isozyme type M2 (PKM2) transported by exosomes affect the metabolic switch in hepatic non-parenchymal cells, that is involved in fibrosis [268]. Another study demonstrated that liver macrophages induce the inactivation of aHSC through the binding of relaxin to miR-30a-5p in their released exosomes [269].

Recently it was reported that exosomes from HCC cells expressing HBV proteins were able to activate HSCs cells promoting their proliferation and fibrosis. Twenty-seven miRNAs were differentially expressed in exosomes from these cells that were involved in signal transduction and inhibition of apoptosis [270].

Similarly to HBV, exosomes derived from HCV-infected hepatocytes are internalized into HSCs and increase the expression of pro-fibrotic markers. These exosomes carry miR-19a and target SOCS3 in HSC, finally activating the STAT3-mediated TGF-β signaling pathway that enhances fibrosis marker genes. Exosomal miR-19a was also increased in sera of chronic HCV patients with fibrosis compared to healthy controls and non-HCV-related liver fibrosis [197]. HCV-infected hepatocytes also release exosomes enriched in miR-192 and deliver them to HSCs. Transported miR-192 increased expression of fibrogenic markers through TGF-β1 upregulation and transdifferentiation of HSCs into myofibroblasts. Inhibition of miR-192 in HCV-replicating hepatocytes inhibited the transdifferentiation of HSCs [194].

Macrophages also release exosomes that are involved in HSC activation. Macrophages treated with lipopolysaccharides (LPSs) express high levels of miR-500 and miR-155-5p. Overexpression or inhibition of miR-500 could accelerate or suppress HSC proliferation and activation as shown in the CCl4-induced liver fibrosis mouse model. miR-500 promotes liver fibrosis by suppressing its targeting gene MFN2, subsequently activating the TGF−β/Smad pathway [271]. Activation of HSCs was also observed after miR-155-5p overexpression [272]. Macrophage-derived exosomes overexpressing miR-103-3p also promote the activation of HSCs by targeting the Krüppel-like factor 4 (KLF4) and can be used as marker of fibrosis, as mentioned before [214]. Interestingly, M2 macrophage-derived exosomes promote activation of HSCs and fibrosis despite the fact that they reduce the immune response. The exosomes derived from M2 macrophages can promote HSC activation. The smooth muscle cell-associated protein 5 (SMAP-5) found in M2 macrophage-derived exosomes is the key protein responsible for HSC activation by modulating autophagy flux. Stimulation of the vitamin D receptor (VDR) reverses its role in M2 macrophage exosomes [273].

Mesenchymal stem cells (MSCs) produce exosomes rich in miR that can ameliorate the progression of liver fibrosis via targeting Smad 4. miR-618 may be transported from MSCs to TGF-β treated HSCs via exosomes inhibiting their viability and migration [274].

Other factors are also involved in HSC activation and proliferation. Increased levels of the lncRNA HEIH was found in serum and exosomes derived from patients with chronic HCV and cirrhosis or HCC [275]. In a co-culture model of hepatocytes and HSCs, it was demonstrated that the lncRNA cytoskeleton regulator RNA (CYTOR) derived from hepatocytes competed with miR-125 for binding to glial cell line-derived neurotrophic factor (GDNF) in HSCs promoting their activation. In addition, downregulation of CYTOR within extracellular vesicles effectively inhibited liver fibrosis in an in vivo model [276]. Finally, after hepatocyte damage, the exosome-mediated activation of TLR3 in HSCs exacerbates liver fibrosis by enhancing IL-17A production by γδ T cells [277].

An overview of the effects of exosomes of different origin in hepatic fibrosis has been recently presented [278].

### 5.3. Role of EVs in Resolution of Viral Fibrosis

Macrophages not only induce activation, but are also directly involved in the resolution of fibrosis as they promote the apoptosis of HSC. Certain macrophage-derived exosomes are crucial in preventing the development of fibrosis [250,279]. IL-6-treated macrophages produce miR-223-rich exosomes to suppress the expression of the profibrotic transcriptional activator with PDZ-binding motif (TAZ) in hepatocytes leading to the impairment of fibrosis advancement [280]. MiR-690 from KCs-derived exosomes repress fibrogenic signals in HSCs and reduce inflammatory signals from recruited BM-derived macrophages [102]. Exosomal miR-411-5p from M2 macrophages inhibits HSCs activation. Mechanistically, exosomal miR-411-5p represses the expression of calmodulin-regulated spectral-associated protein 1 (CAMSAP1) in HSCs, preventing their activation [281]. Relaxin binds to the relaxin receptor expressed by liver macrophages switching them from the profibrogenic to the pro-resolution phenotype. Exosomes rich in miR-30a-5p released from pro-resolution macrophages lead to the relaxin-mediated quiescence of activated HSCs [269].

LSEC-derived EVs suppressed the activation of HSCs, but had no effect on qHSCs. In addition, these EVs suppressed the expression of inflammatory genes in activated KC indicating that LSEC-derived EVs attenuate the fibrogenic phenotype of HSCs and the inflammatory phenotype of KCs [282].

Exosomes originating from other than macrophages cells can also ameliorate liver fibrosis. Serum EVs from healthy humans incorporate higher amounts of miR-34c, -151-3p, -483-5p or -532-5p compared to patients with liver fibrosis. The same mir-RNA profile could inhibit the expression of fibrogenic genes in activated mice HSCs [283]. MSCs-derived exosomes can inhibit fibrosis advancement and collagen deposition through the inhibition of the TGF-β1/SMAD pathway [284]. Human bone MSCs-derived exosomes reduced fibrosis by inhibiting the Wnt/β-catenin preventing HSC activation [285]. Increased autophagy in HSCs attenuated fibrosis by inhibiting the release of fibrotic exosomes [286]. Similarly, exosomes derived from human umbilical cord MSCs reduce liver fibrosis in mice by suppressing the Smad signaling pathway [287]. Liver stem cell-derived exosomes rich in miR-142a-5p, prevented the switch of macrophages toward M1 polarization and facilitated M2 polarization through targeting cathepsin B to suppress fibrosis in the CCl4-induced fibrosis mouse model [288].

Overviews of the role of exosomes in liver fibrosis have been recently published [289,290,291].

### 5.4. Autophagy and EVs in Liver Fibrosis

Autophagy is in fact the recycling system of the cell to use cellular waste and invading pathogens for new synthesis of proteins and lipids. The final step of autophagy is the lysosomal degradation system. However, beyond the recycling role, autophagy is implicated in extracellular release and secretion. Lysosomal exocytosis leads to a fusion of lysosomes with PM and secretion of lysosomal cargo. The autophagy-dependent secretion, is an important mechanism involved in immune signaling [292].

Autophagy is implicated in the regulation of exosome formation. The intersection of exosome biogenesis and autophagy leads to the formation of amphisomes, indicating their synergistic interplay. Parts of the autophagy machinery contribute to exosome biogenesis. Non-autophagic functions of the critical for the autophagy genes ATG5 and ATG16L1 are also critical in exosome biogenesis [293,294].

Autophagy in HSCs alleviates liver fibrosis by inhibiting the release of fibrogenic EVs [286]. However, under limited energy supply in qHSCs, the central to autophagy mTOR pathway is inhibited initiating autophagy to produce ATP, and meet the increased energy requirements during myofibroblast transformation [295]. On the contrary, there is evidence that increased autophagy via inhibition of the mTOR pathway has also the opposite result leading to reduction in HSC activation and attenuation of liver fibrosis [296]. Transformation of HSCs from the quiescent to the activated phenotype consists of two phases, initiation and perpetuation, as mentioned above. Autophagy is involved in both phases. During initiation, impairment of the autophagic flux in nearby cells such as injured hepatocytes, KCs and LSECs release pro-inflammatory and pro-fibrogenic cytokines affecting qHSCs. Normal LSECs is a necessary condition for HSCs to retain the qHSC status [297]. Autophagic dysregulation-associated phase is important for HSCs to respond by entering the perpetuation phase [298]. Once HSCs reach the perpetuation phase, autophagy is downregulated [286,299]. This is necessary for the advancement of fibrosis as stimulation of autophagy is associated with alleviation of liver fibrosis [300]. Furthermore, natural killer cells-derived exosomal miR-223 targets the gene ATG7. The resultant inhibition of autophagy attenuates TGF-β1-induced HSC activation [301].

HBV infection induces autophagy, as shown by using tunicamycin, an N-glycosylation inhibitor and stimulator of endoplasmic reticulum stress that resembles HBV infection. Tunicamycin treatment increased the replication of HBV and release of viral particles and naked capsids [302]. Moreover, low levels of the miRNA miR-192-3p is associated with high levels of HBV DNA in the serum of HBV patients. HBV infection suppressed miR-192-3p through HBx interaction with c-myc leading to increased cellular autophagy. The target of miR-192-3p is the X-linked inhibitor of apoptosis protein (XIAP) mRNA, which is an inhibitor of autophagy. Therefore, HBV-mediated promotion of autophagy is important for HBV replication [303].

HCV is an important model to clarify the connections between autophagy and exosome biogenesis. HCV infection causes upregulation of autophagy, and the release of virus-containing exosomes [192,304]. Deletion of Beclin1 or ATG7, which are factors implicated in autophagy, reduces the release of exosome-associated HCV virus particles [200], indicating that the autophagy machinery is involved in the engulfment of HCV particles into exosomes. Increased autophagosome–lysosome fusion with subsequent degradation decreased the release of HCV particles, indicating that part of HCV particles or its replication mechanism reside within autophagosomes [305]. However, HCV differentially modulates autophagy at different time points. In the early stages of infection, the inhibitor of autophagosome–lysosome fusion Rubicon is upregulated and represses autophagy flux, suggesting that HCV exploits the formation of autophagosomes to assist its replication. Inhibition of Rubicon reduced HCV replication. At a later stage of infection, the UV radiation resistance-associated gene (UVRAG) expression is induced. UVRAC protein activates Beclin1, promoting autophagy. The over-expression of UVRAG facilitated the maturation of autophagosomes and increased HCV replication. Only the HCV NS4B protein is sufficient to induce Rubicon and autophagosomes. The differential induction of Rubicon and UVRAG, indicates that HCV always exploits autophagy for its benefit [306]. The delayed induction of UVRAC in HCV infection possibly reflects altered endosomal trafficking, which favors virus escape via exosomes [307].

Ezetimibe, a drug used to treat hypercholesterolemia, was found to stimulate hepatocyte-derived exosome secretion modulating hepatocyte-macrophage interaction by directly targeting macrophages. These exosomes suppressed the inflammatory activation of macrophages by inhibiting the NLRP3 inflammasome-IL1β pathway. In addition, ezetimibe activated autophagy by activating 5′ AMP-activated protein kinase (AMPK) followed by the transcription factor EB (TFEB) nuclear translocation. These effects justify an investigation of this drug as an antifibrotic agent in fibrotic viral liver diseases [308].

## 6. EVs in Hepatitis-Associated HCC

Approximately 10–25% of chronic HBV infections will progress to HCC. HCC develops at an annual rate of 2–4% of HCV-related cirrhosis. There is a shift from viral to non-viral etiologies of HCC, but this is true only for Europe and North America, but even in the high income countries almost 50% of HCC cases are due to HBV and HCV. Globally, there is only a small reduction in viral etiology from 78% to 70% over the last 30 years [309].

Exosomes are implicated in the induction, progression and metastasis of HCC through RNA transport and protein-mediated cellular intercommunications. Exosomes in HCC cells contain varied miRNAs and promote the growth of liver cancer cells favoring tumor progression [15,310]. MiRNAs regulate the transformational growth factor and activate the kinase-1 (TAK1) pathway [311]. Moreover, exosomes can transport the lncRNA FAL1 into HCC cells to increase cancer cell proliferation, migration and invasion [312]. Exosomes derived from an HCC cell line were internalized by adipocytes, that in turn promote tumor growth, increase angiogenesis and recruit macrophages [313]. It was also reported that exosomes from a highly metastatic cell line can be engulfed by less metastatic HCC cells and promote a more malignant behavior of the recipient cells. Tumor-derived exosomes induce epithelial-to-mesenchymal transformation further promoting HCC invasion [314]. Furthermore, the vacuolar protein sorting-associated protein 4A (Vps4A), which is implicated in intracellular protein trafficking, regulate the release and uptake of exosomes containing both oncogenic and tumor suppressor miRNAs. Reduced expression of Vps4A in HCC can increase HCC progression and metastasis [315]. The loss of miR-320a inhibits the miR-320a-PBX3-MAPK pathway and induce epithelial– mesenchymal transformation and the matrix metalloproteinase 2 (MMP-2) expressions to promote HCC progression and metastasis [316].

The involvement of eVs in HCC development has been particularly investigated in the case of HBV infection. HBV affects the level of cellular miRNA expression in EVs, resulting in an increase in the expression of some miRNAs and a decrease in the expression of others, a change that, promotes the progression of CHB to HCC [153]. In the exosomes released by HBV-related HCC cell lines, over than 40 miRNAs and 200 proteins were significantly dysregulated in the HCC released exosomes compared to normal liver cells. HBV-related HCC cells have characteristic miRNA and protein profiles. These exosomes are CD9-positive, CD63/CD81-negative and are enriched with oncogenic factors and onco-miRNAs [172]. The levels of miR-1269b were also significantly increased in HBV-positive HCC cells compared with HBV-negative cells. HBx facilitates translocation of NF-κB to the nucleus leading to upregulation of CDC40, that promotes, cell proliferation and migration [317]. In another study, immunoregulatory proteins, such as alpha-2-macroglobulin and lactotransferrin, were significantly reduced in exosomes derived from HBV-infected cells [224]. By contrast, the level of valosin-containing protein (VCP) was increased in exosomes secreted from HBV expressing cells and in exosomes from the serum of patients with CHB. VCP is associated with HCC development [318]. These findings indicate that HBV induces infected cells to produce exosomes that can promote the transformation of hepatocytes into cancer cells [224].

EVs can transport both mRNA produced after transcription of viral genes and cellular mRNA encoding proteins which are involved are in the transmission of viral infection [319]. This mechanism is important in carcinogenesis caused by oncoviruses. EVs carry mRNAs to target cells that encode for viral oncoproteins such as mRNAs of HBx in HBV infection [154]. Furthermore, exosomes from HBV-infected liver HCC cells downregulate cell apoptosis when treated with oxaliplatin, increasing thus chemoresistance of these HCC cells. These exosomes also inhibit HCC cell death by activating the chaperone-mediated autophagy (CMA) pathway. The key molecule of CMA, lysosome-associated membrane protein (Lamp2a), was also upregulated [320]. HBx may also interact with the biogenesis pathways of EVs, leading to a change in their protein profile [224]. It was already mentioned that the viral HBcAg protein increases the level of miR-135a-5p in EVs, which has a protective effect against apoptosis and also increases the proliferation of HCC cells and their resistance to chemotherapy [179]. Exosomal miR-122 that suppresses HCC development by binding to the target genes is implicated in proliferation, apoptosis, and angiogenesis in HCC. Reduced miR-122 expression has been reported in patients with HCC [321,322]. It has been shown that HBx promotes the transformation of hepatocytes into HCC cells through the negative regulation of miR-122 expression [323]. MiR-142-3p was significantly increased in HBV-related HCC patients and HBV-infected M1-macrophages. Inhibition of miR-142-3p or overexpression of SLC3A2, an important protein involved in ferroptosis, reversed ferroptosis and inhibited the proliferation, and invasion of HCC cells indicating that miR-142-3p promoted the anti-tumorigenic M1-type macrophage ferroptosis through SLC3A2 [324].

In addition to tumor-promoting miRNAs, HBV-derived exosomes promote HCC development by interfering with the immune response against cancer cells. EVs derived from HBV-infected cells, are endocytosed by monocytes, leading to elevated PD-L1 levels in these cells. This finding explains the increased expression of PD-L1 in the monocytes of patients with CHB [170,325]. PDL-1 inhibits the T cell-mediated immune response against HCC by promoting the exhaustion of T cells after binding to PD-1 on their surface [326]. Additionally, EVs incorporating viral cargo can also negatively affect NK cells. The interaction of EVs carrying HBV cargo with NK cells diminishes their cytotoxic activity, and IFN γ production [152].

The effects of HCV infection is not as extensively studied as for HBV. HCV is also associated with EVs and HCC development, but the relationship has not been fully clarified so far. HCV-related carcinogenesis is mainly mediated by EVs, which induce a favorable microenvironment for transmission of infection and HCC development [327]. As in HBV, high levels of miR-122 found in EVs promote HCV replication [328]. Additionally, increased levels of miR-21 in plasma EVs are associated with pathological features of HCC such as the presence of cirrhosis and tumor staging [220]. An opposite effect on HCC progression is attributed to EVs rich in miR34a. An increase in miR34 in serum of patients with chronic HCV has been observed [329] and has been also correlated with the grade of HCV-related cirrhosis, Patients with mild or moderate fibrosis have lower miR34 level expression in the circulation [330]. Experimental evidence indicate that these findings may in fact be a protective mechanism against HCC. The HCC cell line Huh7.5 was found to express higher levels of miR34a after HCV infection. However, EVs secreted by infected cells with increased miR34a expression, induced apoptosis of non-infected Huh7.5 cells [331] indicating that a cytostatic/cytotoxic effect was induced leading to tumor suppression [332].

**Figure 2:**
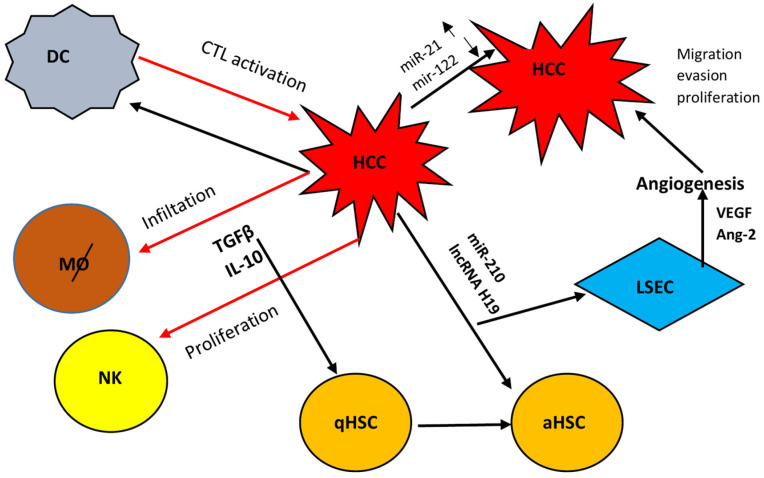
Mechanisms of exosome interference in HCC. HCC-derived exosomes promote proliferation, migration, and invasion of other HCC cells, either directly or indirectly through angiogenesis by activating LSECs to produce exosomes rich in angiogenetic factors. They also promote both stellate cell activation and inhibition of macrophages and NK cells to evade immune response. On the other hand, HCC-derived exosomes may activate dendritic cells to promote the immune response against HCC. For details see text. CTL: Cytotoxic T lymphocytes. DC: dendritic cells. Black arrows indicate activation. Red arrows indicate inhibition.

A simplified diagram of the functions of exosomes in HCC is presented in Figure 2.

## 7. The Therapeutic Use of EVs in Viral Liver Disease

The vast majority of studies is based on experimental data from either cell line experiments or findings from murine models. The few clinical studies were identified in the text.

EVs have a potential therapeutic utilization either as therapeutic targets, or as a drug-related delivery system. EVs originating in several cell populations have been used for this purpose. MSC-derived exosomes are the most frequently tested [333]. Current evidence indicates that the administration of MSCs-derived exosomes is equally effective with administration of intact MSCs [334,335]. Preclinical studies provided evidence that MSC-EVs can reproduce the beneficial effects of MSCs in acute injury including the reduction in cell death and oxidative stress, or the induction of immunomodulation and attenuation of cytokine storm [336]. EVs from MSCs as treatment options are advantageous due to the precise localization in the liver after intravenous injection [285] and most importantly the low immunogenicity due to decreased levels of membrane-bound proteins [337]. Despite these advantages, some problems of MSC-EVs, such as heterogeneity, unequal therapeutic effect, and rapid in vivo clearance are obstacles to their clinical utility. Current efforts to improve the therapeutic potency of MSC-EVs include optimization of culture conditions and modifications of EVs, to facilitate the clinical application of MSC-EV in liver disease [338].

MSC EVs rich in miR-223-3p modulate liver inflammation and fibrosis through inhibition of TGF-β1/Smad2-induced EMT in hepatocytes and repression of the production of pro-inflammatory cytokines and the pro-fibrotic TGF-β1 by Kupffer cells [34]. Exosomes derived from human umbilical cord MSCs (hucMSCs) inhibit both EMT of hepatocytes and collagen production resulting in attenuation of liver fibrosis [339]. Mouse bone marrow MSCs (BM-MSC)-derived rich in miR-223-3p EVs, reduced the liver secretion of pro-inflammatory cytokines and inhibited NLRP3 inflammasome activation in a murine model of autoimmune hepatitis [69]. EVs from human amnion-derived MSCs (AM-MSC) also attenuated LPS-induced inflammation and fibrosis by reducing TNF-α, IL-1β, IL-6, and MCP-1 expression in Kupffer cells and TNF-α expression in hepatic stellate cells in vitro [340].

In the thioacetamide model of chronic liver injury, embryonic MSCs reduced the gene expression of pro-inflammatory cytokines and increased the expression of anti-inflammatory cytokines such as TGF-β1 and IL-10 [341]. Moreover, huc-MSC-derived EVs inhibited the infiltration of neutrophils into the liver and reduced inflammation in the hepatic ischemia-reperfusion injury rat model [342]. Interestingly, EVs from human adipose tissue stem cells significantly improved survival in the D-galactosamine (DGalN) acute liver failure rat model. Increased survival was related to increased levels of the human lncRNA H19 in EVs [343]. BM-MSCs-derived exosomes reduces D-GalN/LPS-induced apoptosis in rat hepatocytes. The expression levels of the pro-apoptotic proteins Bax and cleaved caspase 3 were decreased, and the expression of the anti-apoptotic protein Bcl-2 was increased. Furthermore, autophagy-related markers such as LC3 and Beclin-1 were increased while the use of 3-methyladenine, an autophagy inhibitor, diminished the effects of EVs. It seems therefore, that the reduction in apoptosis is dependent on autophagy regulation [344]. EVs from the mouse macrophage cell line RAW 264.7 pretreated with concanavalin A reduced the production of pro-inflammatory cytokines in a concanavalin A-induced hepatitis mouse model through the expression of miR-122-5p and miR-148a-3p [345].

In view of the above experimental evidence, it was only reasonable to test MSCs-derived EVs in viral hepatitis as they inhibit most cells of the innate and adaptive immunity involved in the pathogenesis of chronic viral hepatitis and cirrhosis, including M1 macrophages, Kupffer cells, Th17 cells, Natural killer T cells (NKT) and neutrophils. This is more interesting as intact MSCs transplanted have disadvantages such as low colonization of the liver and short treatment duration, which affect their therapeutic efficacy [346,347].

Currently drugs are the first line of treatment of chronic HBV infection but limitations do exist [348]. Nucleoside or nucleotide drugs (NUCs) inhibit HBV replication but do not assist the immune system to launch an immune attack against HBV [349]. Exosomes on the other hand may also promote the immune response indicating that they may be used in HBV treatment [350]. Exosomes isolated from a human monocytic cell line stimulated with LPS, evoke a pro-inflammatory response in spleen cells of healthy mice by induction of cytokines [351]. The antiviral effect induced by IFNa can be transported from liver non-parenchymal cells (LNPCs) to HBV-infected hepatocytes. Exosomes from IFN-α-treated LNPCs restore the antiviral status of hepatocytes and the viral replication can be inhibited [126]. Macrophage-derived exosomes transport IFNa-associated miRNAs to HBV-infected hepatocytes through endocytosis and induce antiviral activity against HBV replication and expression [128]. Studies have shown that exosomal miRs-193a-5p, -25-5p, and -574-5p could partly suppress HBV replication. In addition, miR-574-5p could bind to a specific position of the HBV genome and reduce pgRNA and polymerase mRNA levels [183]. Interestingly, the co-localization of HBV envelope proteins with the MVB-associated proteins ALIX and VPS4B inhibition of either of these proteins can block the production and release of the HBV viral envelope [352]. It has also been demonstrated that the complex HBV-miR-3 encoded by HBV, can target its own transcription to inhibit HBsAg, HBeAg, and HBV replication [175]. HBV-miR-3 also enhances the anti-viral effect of IFNa by downregulating SOCS5 in hepatocytes and activating the JAK/STAT. Furthermore, exosomal HBV-miR-3 promoted M1 polarization of macrophages and decreased M2 polarization [176].

Additionally, genetic engineering can be used to modify exosomes. DNA vectors were utilized to generate immunogenic exosomes that can induce cytotoxic T lymphocytes against HBV antigens [353]. Furthermore, the clustered regularly interspaced short palindromic repeats (CRISPR)/associated nuclease (Cas) protein 9 system is a tool that modifies specific genes for degradation [354]. The CRISPER/Cas9 system may be an efficient treatment to eliminate cccDNA in CHB patients [355,356]. It was demonstrated that exosomes derived from CRISPR/Cas9 expressing cells contain functional single-stranded guided RNA (gRNA) and Cas9 protein [357]. These materials can be transported to nearby cells and lead to the destruction of the HBV genome in cells [357,358]. Furthermore, exosomes released from active CD4+ T cells can promote B cell activation and promote the efficiency of the HBsAg vaccine [359]. Exosomes in association with hepatitis B recombinant antigen provoked a humoral response similar to the response to HBsAg solution in mice [351]. Exosomes therefore, may efficiently present antigens and can be used as new vaccines or as adjuvants to provoke antiviral effects.

Although clinical evidence for exosome therapy in HBV is missing, exosome-loaded with drugs may be exploited against HBV in the future. Exosomes derived from the serum of CHB patients treated with Tenofovir Alafenamide (TAF Exo-serum) and TAF-treated macrophages (MP) (Exo-MP-TAF) were tested for antiviral activity in a cell line replicating HBV. Both Exo-serum and Exo-MP-TAF were taken up and exhibited strong antiviral activities reducing the levels of HBsAg, HBeAg, HBV DNA, and most importantly the ccc DNA. The antiviral effects of exosomes were stronger than those of TAF treatment alone. The lncRNA HOTTIP was significantly upregulated in Exo-serum. Further, lncRNA HOTTIP deletion reversed the antiviral effect of Exo-MP-TA indicating that exosomal lncRNA HOTTIP is necessary for the antiviral activity of TAF [360].

Exosomes have been tested for the treatment of chronic HCV as well. Inhibition of the release of EVs significantly reduces viral replication without affecting the viability of the host cells. Therefore, inhibition of EVs release may be a potential antiviral treatment of HCV and other RNA viruses [114]. HucMSCs-derived EVs containing Let-7f, miR-145, miR-199a, and miR-221 inhibited HCV infection [198]. An alternative approach would be the inhibition of exosomal miRNAs that favor HCV infection. HCV RNA incorporated in EVs was closely associated with Ago2, HSP90, and miR-122 leading to stabilization of HCV RNA, as mentioned before [192]. This finding may explain the failure of anti-HCV-receptor antibodies treatment of the virus and suggest as an alternative therapy the inhibition of either miR-122 or HSP90 [361]. Similarly, exosomes released from activated macrophages contain several HCV-resistant miRNAs. These exosomes may be used as treatment of HCV as their uptake by HCV hepatocytes mediates anti-HCV activity by inhibiting HCV replication in hepatocytes [362].

However, there is always the question why to use the difficult and expensive exosomal treatment when certain antiviral drugs have been very efficient to repress inflammation and fibrosis in HCV [363,364], particularly when HCV drug treatment becomes more rationalized [209]. Treatment efforts are fully justified in the final stages of viral disease such as advanced fibrosis, cirrhosis and HCC, where current drugs are mostly ineffective. Again, most studies are based in experimental models. Exosomes from healthy humans derived from umbilical cord, adipose tissue or bone marrow stem cells may be beneficial to patients with liver fibrosis [68,285,287,365]. Liver communications between exosomes and MSCs improve liver fibrosis [287,366]. EVs derived from healthy human serum, rich in miRNAs such as miR-34c, -151-3p, -483-5p, and -532-5p, inhibit the activation of stellate cells and liver fibrosis [283].

EVs released from liver stem cells carry miR-142a-5p, which regulates macrophage polarization through the miR-142a-5p/cathepsin B pathway, leading to improved fibrosis progression accompanied by decreased markers of M1 macrophage polarization and increased markers of M2 polarization, as mentioned before [288]. Moreover, EVs from human plasma, umbilical cord perivascular cells, and amnion epithelial cells attenuate hepatic fibrosis by regulating macrophage function [367,368,369,370].

In vitro, PDGF and its downstream molecule Src homology 2-containing protein tyrosine phosphatase 2 (SHP2) inhibited autophagy and increased HSC-derived EV release. Activation of mammalian target of rapamycin (mTOR) signaling induced the release of MVB-derived exosomes by inhibiting autophagy. Migration of HSCs was promoted by the mTOR-dependent EVs. Selective deletion of SHP2 from HSCs attenuated CCl4-induced liver fibrosis. Moreover, SHP2 was increased in patients with liver cirrhosis [286].

EVs from murine-induced pluripotent stem cells (iPSCs) reduced the expression of pro-fibrogenic genes, including α-SMA, CollagenIα1, and TIMP-1 when administered in models of liver fibrosis [371]. Human BM-MS-derived exosomes ameliorated fibrosis in the CCl4 model of experimental liver fibrosis. Inhibition of the Wnt/β-catenin pathway in HSCs was the underlying mechanism of the anti-fibrotic effect [285].

Additionally, iPSC-derived EVs modulate HSC activation and exert antifibrotic effects, transporting miRs-92a-3p, -10b-5p, and-302-3p with antifibrotic properties. Treatment with iPSC-EVs decrease proliferation and migration of activated HSCs. Human liver stem cells (HLSCs), a multipotent population of adult liver stem cells, may also inhibit HSC activation. Proteomics demonstrated 251 proteins in HLSC-EVs including MMPs, enzymes, and pathways involved in the cytokine, inflammatory, and p53 pathway. The complex protein cargo indicates that HLSC-EVs modulate inflammation and improve liver fibrosis [372]. In addition, HLSC-derived EVs, hepatocyte-derived, BMMSC-derived, and natural killer cell-derived EVs also inhibit HSC activation and liver fibrosis [373,374,375].

Ferroptosis is a form of programmed cell death. The main features include decreased synthesis or depletion of the antioxidant glutathione (GSH) and diminished activity of the glutathione peroxidase 4 (GPX4), the enzyme protecting cell membranes from lipid peroxidation. Reduction in this antioxidant system results in the iron ion-mediated accumulation of lipid peroxides and cellular death [376]. HSCs store significant amounts of ions [377], creating a permissive environment for ferroptosis in these cells. MSC-derived exosomes enhance the stability of the vital-for-ferroptosis protein SLC7A11, activating System xc- and protecting against hepatocyte ferroptosis [378]. Intravenous injection of huCcMSC-derived exosomes enhanced iron elimination in HSCs through the BECN1/GPX4 pathway, suppressed collagen deposition in the liver, ultimately resulting in the attenuation of experimental liver fibrosis [379]. This has been recently confirmed. Treatment with human BMSCs-derived exosomes enriched with miR-26a mimics decreased the level of SLC7A11, induced ferroptosis in HSCs, and attenuated CCL4-induced liver fibrosis [380,381].

Interestingly, the combination of MSCs-derived exosomes with drugs have been used to attenuate fibrosis and cirrhosis [382]. It was demonstrated that the combination of adipose MSC-exosomes with vitamin A derivatives led to the accumulation of exosomes in the periphery of aHSC, which in turn increased the reduction in liver fibrosis. Moreover, even a 10-fold lower dose of vitamin A derivative in exosomes was more effective in attenuation of fibrosis than exosomes alone [382]. Hydroxychloroquine (HCQ) in combination with BM MSC-exosomes, synergistically inhibits autophagy and reduces ECM deposition more efficiently than HCQ or exosomes alone [383].

Exosomes can carry drugs as well. HucMSC-exosomes were used as a drug carrier to transfer obeticholic acid (OCA) and activate the FXR signaling pathway that in turn activates the metalloproteinase 13 (MMP-13), and downregulates TIMP-1. The final result is increased degradation of ECM, and repression of hepatic fibrosis [384]. Quercetin and vitamin A-loaded exosomes derived from AdMSCs were used for the treatment of acute liver failure in mice. Quercetin increased the therapeutic efficacy of exosomes, and vitamin A more efficiently targeted exosomes to the liver. Interestingly, the loaded exosomes decreased the rapid senescence of hepatocytes induced during acute liver injury [385]. In that sense, it should be noted that MSCs-EVs were recently demonstrated to exhibit an anti-senescence function promoting the regeneration of an aged liver by regulating mitophagy [386].

In addition, the exosomes from MSCs loaded with small interfering RNA or antisense oligonucleotides directly target and inhibit STAT3, and could be used to treat fibrosis [387].

An extensive review of the combinations of MSC exosomes with drugs was recently published [388].

However, the most important applications of exosomes as potential medications are in the treatment of HCC, promising innovative therapeutic modalities [389,390]. EVs are important mediators in the complex network of cellular communications within the HCC microenvironment [391,392]. The cargo of EVs contains several bioactive molecules that may interfere with tumor growth [393] and immune evasion of the tumor [394,395], thus inhibiting the tumor progression.

The evidence provided in recent years has proved that exosomes can be loaded with drugs or miRNAs for cancer therapeutic interventions [396,397]. Modified exosomes from AdMSCs were used to transfer miR-199a-3p in HCC cells. Exosomes reduced sorafenib resistance of cancer cells, indicating that exosomes can be used as novel nanocarriers to deliver drugs or molecules to HCC cells [398,399]. Hepatocyte-derived exosomes ameliorate the HCC development through the STAT3 pathway [400]. Exosomes derived from α-fetoprotein (AFP)-expressing dendritic cells (DEXAFP) can induce a strong antigen-specific antitumor immune response mediated by T cells. These exosomes may be used as a cell-free vaccine for HCC immunotherapy [401].

Efforts are made to apply genetically engineered EVs as novel therapeutic strategies against oncoviruses. A new treatment against HCV-related HCC could be the loading of EVs with anti-HCV miRNAs such as let-7f, miR-145, miR-199a, and miR-221 [402]. HBV-related HCC EVs loaded with miR-574-5p may offer an alternative to classic, mostly inefficient drug treatments [183].

The transfer of the exosomal long non-coding RNA *SENP3-EIF4A1* secreted by normal cells to HCC cells increased apoptosis and attenuated the migration of HCC cells, thus inhibiting the progression of HCC [403]. The toll-like receptor 4 (TLR4) is heavily involved in the progression of viral-induced HCC. Therefore, an interesting possibility exists that the use of EVs may impede the progression of HCC through interference with TLR4 [404].

The main therapeutic uses of EVs in liver disease are presented in Table 2.

Currently, the application of exosomes in the treatment of HCC is limited to basic experiments, and further studies are required to explore the applications of exosomes in clinical practice.

Use of EVs as treatment options have recently been reviewed [405,406,407].

## 8. Conclusions

EVs are the third vital mechanism for liver cell intercommunications besides the direct cell-to-cell contact and the secretion of cytokines and chemokines. The recent evidence has proven that they are extremely useful in the clarification of the pathogenesis of viral inflammation and liver fibrosis as they are produced by all cells involved in the induction, progression, or resolution of viral liver diseases. Apart from their pathogenetic role, they may be used as biological markers monitoring the progression of chronic liver diseases. Combinations of miRNAs carried by EVs have prognostic significance in the outcome of viral-induced cirrhosis and HCC. Most importantly, EVs derived mostly from MSCs have been used experimentally in the treatment of HBV- and HCV-related cirrhosis and HCC. Mesenchymal stem cells and their exosomes combined with drugs can effectively improve liver function, reverse hepatic fibrosis, and promote the healing of liver tissue in many animal models of liver fibrosis. Moreover, they inhibit the progression of HCC in in vitro and in vivo models. However, clinical applications are difficult to be implemented due to inherent problems with EV isolation, uneven therapeutic results, and the cost of treatment. Therefore, before advising them as an alternative treatment of viral cirrhosis and HCC, it is necessary to conduct more preclinical studies on the stability and safety of exosomes in viral liver disease. The combined strategy of exosomes and drugs is expected to be a new direction in the treatment of chronic viral liver disease in the near future. In contrast to the use of EVs as therapeutic interventions, their use as biomarkers is difficult to be implemented in the routine clinical practice due to the complexity of their identification.

## Figures and Tables

**Table 1:** EVs biomarkers in viral liver diseases. Arrows: upwards: increased; downwards: decreased.

Disease	Content			Clinical Application	Ref.
Viral Hepatitis	miR-122, -134, -424-3p, 629-5p, -199a miR-301a-3p, -145-5p Exosomal DNA lncRNA NEAT1 miR122 miR-150, -192, -200b,-92a		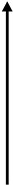	HCV infection HBV treatment responders HBV marker in serum HBV DNA-ve Mortality in acute-on-chronic liver failure -ve correlation with ALT, viral load in HCV Reduced in HBV and HCV	[227] [211] [214] [225] [233] [232]
	
	
		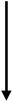			
Liver Fibrosis	mir-155, -19a miR-214 miR-21 miR-103-P mir-122 miR-192, -200b, -92a, -150 miR-29, -143, -223, -21, -374, -93	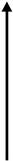		HCV LF HCV LF +ve correlation with HCV fibrosis Degree of HBV fibrosis -ve correlation with HCV fibrosis Early fibrosis HBV-HCV Gradual reduction as HBV LF increases	[217,231] [228] [233] [233] [212]


		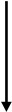			
HCC	hsa-circ002862, hsa-circ0070396 miR-483-5p/ALCAM 29a,-29c,-133a,-143,-145,-192,-505 miR-21 miR-18a, -221, -222, -224 mir-125b miR-101, -106b, -122, -125		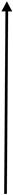	HBV-related HCC Invasion-metastasis of HBV-HCC Accurate diagnosis HCC Diagnosis HBV-related HCC Diagnosis Diagnosis of HBV-related HCC Detection, prognosis	[243,244] [247] [238] [235] [236] [240,241] [236]


			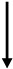		

The use of EVs as biochemical markers has been recently published [18].

**Table 2:** Treatment strategies with MSc-derived EVs in chronic liver disease. Data are based on experimental models of liver disease, mostly on CCl4 models.

Source	Contents	Effect	Ref.
Non-parenchymal liver cells Hepatocytes Human serum Liver stem cells	miRs-193a-5p, -25-5p, and -574-5p HBV-miR-3 miR-34c, -151-3p, -483-5p, and -532-5p, miR-142a-5p	Suppress HBV replication Suppress HBV replication Inhibit activation of HSCs Reduced LF-increased M2 polarization	[182] [174,175] [282] [287]
Ad-MSCs	miR-199a-3p miR-122 human lncRNA H19	Reduced sorafenib resistance in HCC Reduces chemo resistance in HCC. Inhibits HCC progression Increased survival in ALF	[397] [319,396] [341]
Normal Human cells	lncRNA SENP3-EIF4A1	Stimulation of apoptosis in HCC	[401]
Induced pluripotent stem cells	miRs-92a-3p, -10b-5p and -302-3p **---**	Reduces LF Reduced the expression of pro-fibrogenic genes	[370,371] [369]
α-fetoprotein expressing dendritic cells (DEXAFP)	**---**	Antigen-specific antitumor immune response mediated by T cells	[399]
Huc-MSCs	Let-7f, miR-145, miR-199a, -221 miR-451a	Inhibition of HCV infection Reduces chemo-resistance, proliferation, and invasion in HCC	[197] [94]
BM-MSCs	**---** miR-223-3p EVs,	Inhibition of Wnt/β-catenin Reduction in the liver secretion of pro-inflammatory cytokines; inhibition of NLRP3 inflammasome.	[284] [69]

Ad-MSCs: adipose-MSC-derived EVs; Huc-MSCs: human umbilical cord MSC-derived EVs; BM-MSCs: Bone marrow-MSC-derived EVs.

## Data Availability

Not applicable.
